# Supplementary material for: New insights into macrophage polarization and its prognostic role in patients with colorectal cancer liver metastasis
Source: BJC Rep. 2024 Apr 26;2:37. doi: 10.1038/s44276-024-00056-8 (PMC11523988; doi:10.1038/s44276-024-00056-8)
Supplement: Supplementary file 1 — Supplementary Tables [file 44276_2024_56_MOESM1_ESM.docx]

**Supplementary Tables:**

**Table S1: Phenotypes of the macrophage and adaptive T cell subtypes in the multiplex immunofluorescence panel**

| **Cell type** | **Phenotype** |
| --- | --- |
| Malignant cells | CK+ |
| Total macrophages | CD68+ |
| M1 macrophage | CD68+CD86+CD163-CD206-Arg1- |
| M2 macrophage  TGFβ expressing macrophages | CD68+CD163+MRP8-14-CD86-  CD68+ TGFβ+ |
| pSMAD3 expressing macrophages | CD68+ pSMAD3+ |
| FOXP3 expressing macrophages | CD68+ FOXP3+ |
| T cells | CD3 |
| Helper T cells | CD3+CD4+ |
| Cytotoxic T cells | CD3+CD8+ |
| Regulatory T cells | CD3+FOXP3+ |
| TGFβ expressing T cells | CD3+ TGFβ |
| TGFβ expressing Helper T cells | CD3+CD4+ TGFβ |
| TGFβ expressing Cytotoxic T cells | CD3+CD8+ TGFβ |
| TGFβ expressing Regulatory T cells | CD3+FOXP3+ TGFβ |
| pSMAD3 expressing T cells | CD3+ pSMAD3+ |
| pSMAD3 expressing Helper T cells | CD3+CD4+ pSMAD3+ |
| pSMAD3 expressing Cytotoxic T cells | CD3+CD8+ pSMAD3+ |
| pSMAD3 expressing Regulatory T cells | CD3+FOXP3+ pSMAD3+ |

**Table S2: Antibodies clones of markers used in the multiplex immunofluorescence panel**

| **Antibody** | **Clone** | **Dilution** | **Company** | **OPAL Dye spectra (nm)** |
| --- | --- | --- | --- | --- |
| CK | AE1/AE3 | 1:25 | Dako | 540 |
| CD68 | PG-M1 | 1:25 | Abcam | 520 |
| CD163 | 10D6 | 1:100 | Leica Biosystems | 690 |
| CD86 | E2G8P | 1:100 | Cell Signaling Technology | 620 |
| CD206 | PA5-83759 | 1:100 | Thermo Fisher | 570 |
| PD-L1 | E1L3N | 1:100 | Cell Signaling Technology | 480 |
| ARG-1 | D4E3M^M^ | 1:200 | Cell Signaling Technology | 650 |
| MRP8-14 | 27E10 | 1:50 | Abcam | 780D |
| CD3 | D7A6E | 1:100 | Cell Signaling Technology | 480 |
| CD4 | EPR6855 | 1:100 | Abcam | 690 |
| CD8 | C8/144B | 1:25 | Abcam | 520 |
| FOXP3 | D2W8E | 1:50 | Cell Signaling Technology | 570 |
| TGFβ | EPR21143 | 1:50 | Abcam | 620 |
| pSMAD3 | EP823Y | 1:200 | Abcam | 780D |

**Table S3: Distribution of macrophages and adaptive T cells in CLM.**

|  |  |  |
| --- | --- | --- |
| **Cell Phenotype**  **n/mm^2^** | **Median density (range)** | **Mean**  **density** |
| Total CD68+ | 45 (2-273) | 61 |
| CD68+pSMAD3+ | 2 (0-93) | 5 |
| CD68+TGFB+ | 1 (0-29) | 3 |
| CD68+ FOXP3+ | 0 (0-16) | 1 |
| CD68+CD163+ (M2) | 1 (0-97) | 4 |
| CD68+CD86+ (M1) | 0 (0-14) | 1 |
| Total CD3+ | 17 (0-389) | 35 |
| CD3+CD4+ | 2 (0-259) | 15 |
| CD3+CD8+ | 1 (0-49) | 5 |
| CD3+FOXP3+ | 0 (0-6) | 1 |
| CD3+CD4+FOXP3+ | 0 (0-5) | 0 |
| CD3+pSMAD3+ | 0 (0-18) | 1 |
| CD3+CD4+pSMAD3+ | 0 (0-5) | 0 |
| CD3+CD8+pSMAD3+ | 0 (0-10) | 0 |
| CD3+FOXP3+pSMAD3+ | 0 (0-1) | 0 |
| CD3+CD4+FOXP3+pSMAD3+ | 0 (0-1) | 0 |
| CD3+TGFB+ | 1 (0-48) | 3 |
| CD3+CD4+TGFB+ | 0 (0-3) | 0 |
| CD3+CD8+TGFB+ | 0 (0-2) | 0 |
| CD3+FOXP3+TGFB+ | 0 (0-1) | 0 |
| CD3+CD4+FOXP3+TGFB+ | 0 (0) | 0 |

| **Table S4: Univariate and multivariate Cox proportional hazard model overall survival analysis for clinicopathologic features and adaptive T cell and macrophage phenotypes in entire CLM cohort (n=105)** | | | | | | | |
| --- | --- | --- | --- | --- | --- | --- | --- |
|  |  | **Univariate** | | | **Multivariate** | | |
| **Factor** |  | **HR** | **95% CI** | **p value** | **HR** | **95% CI** | **p value** |
| Age(year) |  | 1.01 | 0.988 – 1.032 | 0.373 |  |  |  |
| Gender |  | 0.80 | 0.505 – 1.254 | 0.325 |  |  |  |
| Primary tumor location | | 1.27 | 0.766 – 2.105 | 0.353 |  |  |  |
| Primary tumor T stage (T3-T4) | | 1.67 | 0.722 – 3.852 | 0.232 |  |  |  |
| Primary nodal status (N1) | | 1.75 | 1.011 – 3.018 | **0.046** | 2.01 | 1.145 – 3.526 | **0.015** |
| Extrahepatic metastases | | 1.27 | 0.609 – 2.651 | 0.523 |  |  |  |
| Synchronous liver metastasis | | 1.19 | 0.751 – 1.871 | 0.466 |  |  |  |
| Preoperative CEA level (> 5 ng/mL) | | 0.95 | 0.600 – 1.487 | 0.806 |  |  |  |
| Prehepatectomy neoadjuvant chemotherapy | | 0.92 | 0.580 – 1.444 | 0.703 |  |  |  |
| Major hepatectomy (Couinaud >=3) | | 1.03 | 0.637 – 1.675 | 0.895 |  |  |  |
| Post hepatectomy chemotherapy | | 1.56 | 0.909 – 2.667 | 0.107 |  |  |  |
| Diameter of largest liver metastasis (>5 cm) | | 0.96 | 0.537 – 1.723 | 0.897 |  |  |  |
| Number of liver metastasis | | 1.28 | 0.815 – 2.024 | 0.281 |  |  |  |
| Liver resection margin | | 1.90 | 0.901 – 3.983 | **0.092** | 2.21 | 1.037 – 4.699 | **0.040** |
| **Cell phenotype** | |  |  |  |  |  |  |
|  | CD68+ | 0.73 | 0.460 – 1.157 | 0.180 |  |  |  |
|  | CD68+pSMAD3+ | 1.15 | 0.707 – 1.871 | 0.573 |  |  |  |
|  | CD68+FOXP+ | 1.22 | 0.774 – 1.919 | 0.393 |  |  |  |
|  | CD68+TGFb+ | 1.32 | 0.795 – 2.190 | 0.284 |  |  |  |
|  | CD68+CD86+ (M1) | 0.96 | 0.585 – 1.584 | 0.880 |  |  |  |
|  | CD68+CD163+ (M2) | 0.74 | 0.470 – 1.177 | 0.206 | 0.62 | 0.386 – 0.997 | **0.049** |
|  | CD3+ | 0.92 | 0.585 – 1.453 | 0.726 |  |  |  |
|  | CD3+pSMAD3+ | 1.33 | 0.835 – 2.131 | 0.228 |  |  |  |
|  | CD3+pFOXP3+ | 1.24 | 0.390 – 3.964 | 0.712 |  |  |  |
|  | CD3+TGFb+ | 0.85 | 0.535 – 1.336 | 0.473 |  |  |  |
|  | CD3+CD4+ | 0.85 | 0.542 – 1.346 | 0.496 |  |  |  |
|  | CD3+CD4+pSMAD3+ | 0.96 | 0.542 – 1.689 | 0.878 |  |  |  |
|  | CD3+CD4+FOXP3+ | 1.41 | 0.854 – 2.322 | 0.180 |  |  |  |
|  | CD3+CD4+TGFb+ | 0.93 | 0.404 – 2.155 | 0.872 |  |  |  |
|  | CD3+CD8+ | 0.90 | 0.568 – 1.423 | 0.651 |  |  |  |
|  | CD3+CD8+pSMAD3+ | 1.17 | 0.686 – 1.991 | 0.566 |  |  |  |
|  | CD3+CD8+TGFb+ | 1.20 | 0.656 – 2.187 | 0.557 |  |  |  |
| Abbreviations: HR, hazard ratio: CI, confidence interval: CEA, carcinoembryonic antigen: CLM, colorectal liver metastasis:  Factors with a threshold p value < 0.10 were selected for the final model. | | | | | | | |

**Table S5: Univariate and multivariate Cox proportional hazard model overall survival analysis for clinicopathologic features and macrophage phenotypes in CLM without preoperative chemotherapy (n=49)**

|  |  | **Univariate** | | | **Multivariate** | | | |
| --- | --- | --- | --- | --- | --- | --- | --- | --- |
| **Factor** |  | **HR** | **95% CI** | **p value** | | **HR** | **95% CI** | **p value** |
| Age (year) |  | 1.01 | 0.982 – 1.041 | 0.457 |  | |  |  |
| Gender |  | 1.04 | 0.523 – 2.058 | 0.916 |  | |  |  |
| Primary tumor location |  | 1.18 | 0.545 – 2.541 | 0.678 |  | |  |  |
| Primary tumor T stage (T3-T4) | | 1.64 | 0.574 – 4.710 | 0.355 |  | |  |  |
| Primary nodal status (N1) | | 1.30 | 0.605 – 2.801 | 0.499 |  | |  |  |
| Extrahepatic metastasis, | | 1.83 | 0.632 – 5.272 | 0.266 |  | |  |  |
| Synchronous liver metastasis | | 0.95 | 0.467 – 1.917 | 0.878 |  | |  |  |
| Preoperative CEA level (> 5 ng/mL) | | 0.72 | 0.355 – 1.459 | 0.361 |  | |  |  |
| Post hepatectomy chemotherapy, yes | | 1.02 | 0.488 – 2.143 | 0.954 |  | |  |  |
| Diameter of largest liver metastasis (>5 cm) | | 1.27 | 0.587 – 2.733 | 0.547 |  | |  |  |
| Number of liver metastasis | | 2.24 | 1.116 – 4.512 | **0.023** | 2.41 | | 1.194 – 4.862 | **0.014** |
| Liver resection margin |  | 2.77 | 0.811 – 9.431 | 0.104 |  | |  |  |
| **Cell phenotype** |  |  |  |  |  | |  |  |
|  | CD68+ | 0.81 | 0.408 – 1.586 | 0.530 |  | |  |  |
|  | CD68+pSMAD3+ | 0.57 | 0.278 – 1.148 | 0.114 |  | |  |  |
|  | CD68+TGFb+ | 0.58 | 0.284 – 1.167 | 0.126 |  | |  |  |
|  | CD68+FOXP+ | 0.96 | 0.455 – 2.014 | 0.908 |  | |  |  |
|  | CD68+CD163+ (M2) | 0.56 | 0.280 – 1.098 | 0.091 | 0.51 | | 0.256 – 1.009 | 0.053 |
|  | CD68+CD86+ (M1) | 0.83 | 0.388 – 1.707 | 0.644 |  | |  |  |
| Abbreviations: HR, hazard ratio: CI, confidence interval: CEA, carcinoembryonic antigen: CLM, colorectal liver metastasis Factors with a threshold p value < 0.10 were selected for the final model. | | | | | | | | |

**Table S6: Univariate and multivariate Cox proportional hazard model overall survival analysis for clinicopathologic features and macrophage phenotypes in CLM with preoperative chemotherapy (n=56)**

|  |  | **Univariate** | | | **Multivariate** | | |
| --- | --- | --- | --- | --- | --- | --- | --- |
| **Factor** |  | **HR** | **95% CI** | **p value** | **HR** | **95% CI** | **p value** |
| Age (year) |  | 1.00 | 0.968 – 1.036 | 0.940 |  |  |  |
| Gender |  | 0.69 | 0.370 – 1.284 | 0.241 |  |  |  |
| Primary tumor right vs. left colon/rectum) location |  | 1.19 | 0.591 – 2.377 | 0.632 |  |  |  |
| Primary tumor T stage (T3-T4) | | 1.51 | 0.364 – 6.277 | 0.570 |  |  |  |
| Primary nodal status (N1) | | 2.15 | 0.974 – 4.733 | 0.058 | 2.04 | 0.904 – 4.580 | 0.086 |
| Extrahepatic metastasis, yes | | 1.02 | 0.361 – 2.866 | 0.974 |  |  |  |
| Synchronous liver metastasis | | 1.59 | 0.792 – 3.193 | 0.192 |  |  |  |
| Preoperative CEA level (> 5 ng/mL) | | 1.06 | 0.562 – 1.994 | 0.861 |  |  |  |
| Post hepatectomy chemotherapy | | 3.18 | 1.215 – 8.324 | **0.018** | 3.38 | 1.241 – 9.206 | **0.017** |
| Diameter of largest liver metastasis (>5 cm) | | 0.67 | 0.260 – 1.700 | 0.394 |  |  |  |
| Number of liver metastasis | | 0.97 | 0.514 – 1.818 | 0.917 |  |  |  |
| Liver resection margin | | 1.65 | 0.638 – 4.250 | 0.303 |  |  |  |
| Major pathological response* | | 1.39 | 0.728 – 2.635 | 0.321 |  |  |  |
| **Cell phenotypes** |  |  |  |  |  |  |  |
|  | CD68+ | 0.68 | 0.352 – 1.297 | 0.239 |  |  |  |
|  | CD68+pSMAD3+ | 2.46 | 1.304 – 4.619 | **0.005** | 3.06 | 1.603 – 5.827 | **0.001** |
|  | CD68+TGFb+ | 1.25 | 0.669 – 2.351 | 0.480 |  |  |  |
|  | CD68+FOXP+ | 1.43 | 0.736 – 2.778 | 0.292 |  |  |  |
|  | CD68+CD163+ (M2) | 0.90 | 0.475 – 1.698 | 0.741 |  |  |  |
|  | CD68+CD86+ (M1) | 1.10 | 0.567 – 2.137 | 0.776 |  |  |  |
| Abbreviations: HR, hazard ratio: CI, confidence interval: CEA, carcinoembryonic antigen: CLM, colorectal liver metastasis *Data of major pathological response was missing in 3 patients. Factors with a threshold p value < 0.10 were selected for the final model. | | | | | | | |
